# Striatal Synaptic Dysfunction and Hippocampal Plasticity Deficits in the Hu97/18 Mouse Model of Huntington Disease

**DOI:** 10.1371/journal.pone.0094562

**Published:** 2014-04-11

**Authors:** Karolina Kolodziejczyk, Matthew P. Parsons, Amber L. Southwell, Michael R. Hayden, Lynn A. Raymond

**Affiliations:** 1 Department of Psychiatry, Brain Research Centre, University of British Columbia, Vancouver, British Columbia, Canada; 2 Centre for Molecular Medicine and Therapeutics, Child and Family Research Institute, University of British Columbia, Vancouver, British Columbia, Canada; Karolinska Inst, Sweden

## Abstract

Huntington disease (HD) is a fatal neurodegenerative disorder caused by a CAG repeat expansion in the gene (*HTT*) encoding the huntingtin protein (HTT). This mutation leads to multiple cellular and synaptic alterations that are mimicked in many current HD animal models. However, the most commonly used, well-characterized HD models do not accurately reproduce the genetics of human disease. Recently, a new ‘humanized’ mouse model, termed Hu97/18, has been developed that genetically recapitulates human HD, including two human *HTT* alleles, no mouse *Hdh* alleles and heterozygosity of the HD mutation. Previously, behavioral and neuropathological testing in Hu97/18 mice revealed many features of HD, yet no electrophysiological measures were employed to investigate possible synaptic alterations. Here, we describe electrophysiological changes in the striatum and hippocampus of the Hu97/18 mice. At 9 months of age, a stage when cognitive deficits are fully developed and motor dysfunction is also evident, Hu97/18 striatal spiny projection neurons (SPNs) exhibited small changes in membrane properties and lower amplitude and frequency of spontaneous excitatory postsynaptic currents (sEPSCs); however, release probability from presynaptic terminals was unaltered. Strikingly, these mice also exhibited a profound deficiency in long-term potentiation (LTP) at CA3-to-CA1 synapses. In contrast, at 6 months of age we found only subtle alterations in SPN synaptic transmission, while 3-month old animals did not display any electrophysiologically detectable changes in the striatum and CA1 LTP was intact. Together, these data reveal robust, progressive deficits in synaptic function and plasticity in Hu97/18 mice, consistent with previously reported behavioral abnormalities, and suggest an optimal age (9 months) for future electrophysiological assessment in preclinical studies of HD.

## Introduction

Huntington disease (HD) is a fatal autosomal dominant neurodegenerative disorder caused by a CAG repeat expansion in the gene (*HTT*) encoding the huntingtin protein [1]. Expansion of the CAG beyond 36 repeats produces a mutated huntingtin protein (muHTT) with an expanded polyglutamine (polyQ) tract in its N-terminal region. Expression of muHTT results in profound disruption of a wide variety of cellular processes (reviewed in: [2]), including synaptic transmission and plasticity (reviewed in: [3]–[5]). It is of a great interest to determine whether therapeutics designed to delay or slow progression of HD can prevent or reverse these detrimental cellular changes, and how early in disease development they should be applied to exert their full beneficial effects.

A recent and promising approach to the treatment of HD utilizes antisense oligonucleotide (ASO) technology to knock down expression levels of muHTT. ASO administration has been employed to effectively silence the expression of a variety of genes of interest and relies on a specific design and efficient delivery of short, synthetic, modified nucleic acids. A number of preclinical and clinical studies, targeting different neurological and non-neurological disorders, have demonstrated ASO efficiency with no severe adverse effects (reviewed in: [6], [7]).

ASOs targeted to muHTT, designed by exploiting the presence of single nucleotide polymorphisms (SNPs), have proven to be potent and selective in cell culture and the BACHD mouse model [8]. However, studying the effectiveness of anti-muHTT ASO therapy requires preclinical testing in an animal model that fully recapitulates the genetics of the disease and displays behavioral, cellular and synaptic deficits typical for HD. The most commonly used, well-characterized HD models do not accurately reproduce human HD genetics. Specifically, they express either mouse HTT with expanded polyQ repeats (knock-in models) or human muHTT on a mouse wild-type HTT background (fragment and full-length transgenic models; reviewed in: [9], [10]). To overcome these obstacles, a new “humanized” mouse model termed Hu97/18 has been developed, and behavioral and neuropathological tests have shown it recapitulates many aspects of HD [11]. Apart from changes in motor function and reduced motor learning at 2 months of age, Hu97/18 mice show additional cognitive deficits as early as 6 months, progressing to further deficits at 9 months; these include a decline in both spatial and object recognition memory performance. Furthermore, striatal and cortical volume is significantly decreased in these animals by 12 months of age [11]. This model is unique in that it expresses only the human HD gene, heterozygous for the HD mutation (with an expanded CAG of 97 repeats), on a mouse Huntingtin *null* (*Hdh^-/-^*) background [11], and therefore provides a valuable tool to evaluate the efficacy of selective muHTT ASO therapies.

As mentioned, HD is associated with a host of cellular and synaptic alterations and it is important for preclinical studies to determine whether a therapeutic strategy can successfully prevent or reverse such changes. While many electrophysiological signatures have been described previously in other animal models of HD [4], none have been reported for the Hu97/18 mouse model. In the present study, we describe electrophysiological changes in the striatum and hippocampus of the Hu97/18 mice. We provide evidence for robust deficits in synaptic function and plasticity that should prove essential for future assessment of the effectiveness of ASO treatment and other candidate therapeutics in preclinical studies of HD.

## Materials and Methods

### Ethics statement

This study was carried out in strict accordance with the requirements of Canadian Council on Animal Care (CCAC). The study was approved by the University of British Columbia Animal Care Committee, under protocol A11-0012. All efforts were made to minimize animal suffering.

### Transgenic mice and brain slice preparation

Transgenic Hu18/18 and Hu97/18 mice expressing full-length human huntingtin with 18 CAG repeats (Hu18/18) or with 18 and 97 CAG repeats (Hu97/18) carried on the yeast artificial chromosome (YAC) or bacterial artificial chromosome (BAC) transgene, respectively, on the *Hdh^-/-^*background (strain FVB/N), were generated and maintained at the animal care facility in the Centre for Molecular Medicine and Therapeutics [11] and then transferred to the University of British Columbia Animal Research Unit approximately 2 weeks prior to experimentation. Experiments were conducted either on mice aged 9–9.5 months (9-month old group), 6–7 months (6-month old group) or 3–3.5 months (3-month old group). All reported n values represent the number of cells or slices recorded. At least 3 (typically 5 or more) animals per genotype were used for each set of experiments. All data were obtained from male mice with the exception of a small number of female mice that were used in the 6-month group. The data obtained from females at this age did not differ from the males and was therefore pooled together.

To obtain acute corticostriatal slices, the animals were killed by decapitation following deep halothane vapour anaesthesia (in accordance with the UBC Animal Care Committee, protocol A11-0012, and the Canadian Council on Animal Care). The brain was immediately removed and immersed in ice-cold oxygenated slicing medium, composed of (in mM): NaCl 125, NaHCO_3_ 25, NaH_2_PO_4_ 1.25, KCl 2.5, CaCl_2_ 0.5, MgCl_2_ 4, D-glucose 10 (gassed with 95% O_2_/5% CO_2_), pH 7.3–7.4, 300–315mOsm. Coronal slices 300 µm thick were cut on a vibratome (Leica VT1200S) and placed in a holding chamber containing oxygenated artificial cerebrospinal fluid (ACSF) at 34–36°C. The ACSF composition was the same as the slicing medium except with 2 mM CaCl_2_ and 1 mM MgCl_2_. After 30–45 min, slices were transferred to room temperature (RT) and kept until 8 h post-slicing. For all hippocampal experiments, slices were obtained as above but in the transverse plane at 400 µm thickness. Sections were given 30–45 minutes at 34–36°C followed by an additional 30–45 minutes at RT to recover. Hippocampal slices were used for a maximum of 6 hours following recovery.

### Electrophysiology

Much of the electrophysiology was performed as described previously [12]. Slices were continuously perfused (∼2–4 ml/min; RT) with oxygenated ACSF containing picrotoxin (50–100 µM; PTX, Tocris Bioscience, Bristol, UK), glycine (10 µM; Sigma-Aldrich, MO, USA) and strychnine (2 µM; Tocris Bioscience, Bristol, UK). Glycine and strychnine were omitted for all hippocampal experiments and PTX was also omitted for LTP experiments. All signals, unless stated otherwise, were filtered at 10 kHz, digitized at 10 kHz and analysed in Clampfit10.2 (Axon Instruments, CA, USA). Whole cell patch-clamp recordings were performed in voltage-clamp or current-clamp mode. For the experiments in voltage-clamp mode (paired-pulse ratio and evoked stimulation), the internal solution consisted of (in mM): caesium methanesulphonate 130, CsCl 5, NaCl 4, MgCl_2_ 1, EGTA 5, HEPES 10, QX-314 5, Na_2_GTP 0.5, Na_2_-phosphocreatine 10,spermine 0.1 and MgATP 5, pH 7.3, 280–290mOsm. For the experiments in current-clamp mode, as well as spontaneous excitatory postsynaptic currents (sEPSCs) and miniature EPSC (mEPSCs) recordings, the internal solution consisted of (in mM): K-gluconate 145, MgCl_2_ 1, HEPES 10, EGTA 1, MgATP 2, Na_2_GTP 0.5, pH 7.3, 280–290mOsm. Pipette resistance (Rp) was 3–5 MΩ. Series resistance (Rs) was *<*30 MΩ and uncompensated; the data were not included in the analysis if Rs changed by *>*20% by the end of the experiment. For LTP experiments, glass electrodes (1–2 MΩ) were filled with ACSF and used to stimulate the Schaffer collateral pathway and record field excitatory postsynaptic potentials (fEPSPs) in CA1 stratum radiatum. Electrical stimulation (0.1 ms pulses) was increased to generate the maximal response and then reduced to the stimulation intensity that produced 30–40% of the maximal response. Responses were elicited every 20 s for at least 10 minutes prior to LTP induction. High frequency stimulation (HFS; 100 Hz for 1 s×3, 10 s inter-train interval) was applied, and responses were again recorded at 20 s intervals for 60 minutes thereafter.

sEPSCs and mEPSCs were filtered at 1 kHz, digitized at 10 kHz and analysed with Clampfit10.2 event analysis function with a detection threshold set at -8pA (SPNs) or -6pA (CA1) while recording at a holding potential of ­70 mV. Decay time was measured using single exponential fitting by Clamfit10.2 event analysis function.

Evoked EPSCs (eEPSCs) were elicited by intrastriatal electrical stimulation through a glass micropipette filled with ACSF (Rp = 2–5 MΩ) placed 150–200 µm dorsal to the recorded cell. The paired-pulse ratio (PPR) was recorded at V_h_ of -70 mV, and when performed with different stimulation intensities (50–500 µA), a 50 ms interval was applied. NMDA peak current was measured at +40 mV 40 ms after the initial peak response (to eliminate the possibility of contamination by the fast-decaying AMPA currents, [13]). Evoked responses (at both potentials) were averages of three responses recorded at the same stimulation intensity.

### Data analysis and statistics

Data were analysed using Clampfit10.2 (Axon Instruments, CA, USA), Microsoft Excel (Microsoft Corp., CA, USA) and Graphpad Prism5 (Graphpad Software, CA, USA). Data are presented as mean ±s.e.m. Significance was assessed with 2-tailed unpaired Student’s t-tests with Welch correction or with two-way ANOVA followed by Bonferroni’s *post hoc* test; P-values <0.05 were considered significant.

## Results

### Cellular and synaptic properties of striatal spiny projection neurons in 9 month-old Hu18/18 and Hu97/18 mice

**Figure 1:**
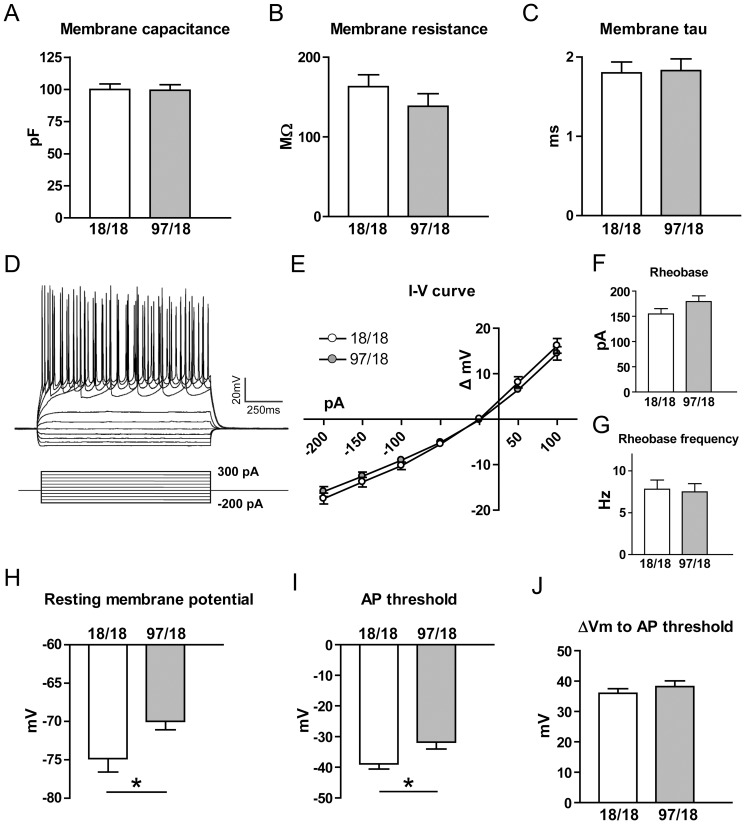
Alterations in basic membrane properties of Hu97/18 SPNs. (A–C): Membrane capacitance (A), membrane resistance (B), and membrane time constant (C) did not differ between Hu18/18 and Hu97/18 SPNs. Membrane capacitance measurement was pooled from the experiments with potassium-based and caesium-based internal solutions (Hu18/18 n = 46, Hu97/18 n = 44), while membrane resistance and tau were measured with potassium-based internal solution only (Hu18/18 n = 21, Hu97/18 n = 17). (D – J): Cells were patch-clamped with potassium-based solution, and membrane voltage changes in response to the injected current were recorded and analysed. (D) Representative I–V response of a Hu18/18 SPN to current injection (50pA increments from -200pA, 1 s each). (E) I–V curves showed no difference between Hu97/18 and Hu18/18 SPNs. (F) Rheobase and (G) rheobase frequency were not different between the genotypes. (H) Hu97/18 had a lower resting membrane potential than Hu18/18 SPNs (p = 0.03). (I) Action potential (AP) threshold was more depolarized in Hu97/18 SPNs (p = 0.02). (J) The change in membrane voltage from resting potential to AP threshold was not different between Hu18/18 and Hu97/18 SPNs. For the experiments in D–J, Hu18/18 n = 11 and Hu97/18 n = 12. *p<0.05 (unpaired Student’s t-test).

Hu97/18 mice develop motor learning deficits as early as at 2 months of age, but memory impairments arise at 6 months of age and become fully manifest by 9 months [11]. Therefore, we began by using 9 month-old animals to assess the electrophysiological changes in the striatum and hippocampus. We identified striatal SPNs in the CPu of mouse striata as described previously [12] and first assessed passive membrane properties in voltage clamp mode (V_h_ = -70 mV). Using a potassium-based internal solution, the membrane capacitance, membrane resistance and membrane tau were not significantly different between genotypes (Fig. 1A–C). Cell firing characteristics were assessed in current clamp by recording membrane voltage changes in response to injected current steps (50pA increments starting from -200pA, 1 s each; Fig. 1D). The I–V curves were similar between the two genotypes (Fig. 1E), as was rheobase (Fig. 1F) and rheobase frequency (Fig. 1G), suggesting no changes in SPN excitability. Notably, Hu97/18 SPNs showed a significant depolarization of the resting membrane potential (p = 0.03, Fig. 1H); this change might be expected to result in an increased steady-state sodium channel inactivation. Consistent with this prediction, the action potential threshold was indeed elevated in Hu97/18 SPNs (p = 0.02, Fig. 1I) and the average change of membrane voltage from resting membrane potential to AP threshold was not different between the genotypes (Fig. 1J). Together, these results indicate that despite small changes in SPN membrane properties, the excitability of SPNs is similar for the two genotypes.

**Figure 2:**
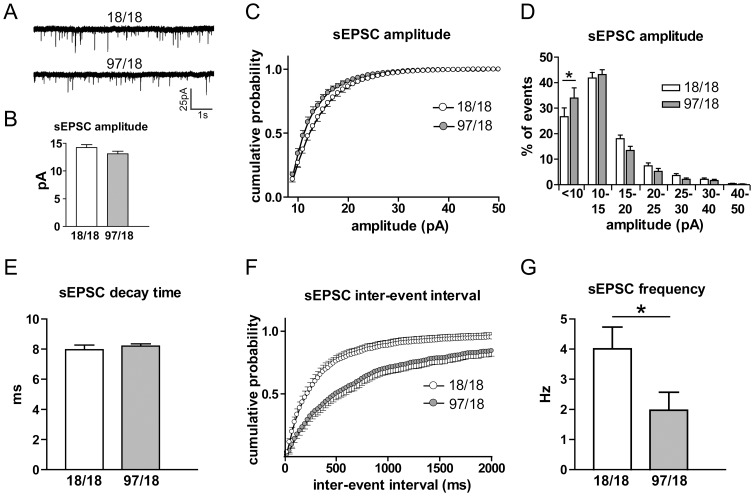
sEPSC amplitude and frequency change in Hu97/18 SPNs. Cells were patch-clamped with a potassium-based internal solution at V_h_ = -70 mV and sEPSCs were recorded. (A) Representative sEPSC traces for Hu18/18 (top) and Hu97/18 SPNs (bottom). (B) The average sEPSC amplitude of Hu18/18 and Hu97/18 cells (difference did not reach significance, p = 0.12). (C) Cumulative probability showed a decrease in sEPSC amplitude in Hu97/18 SPNs (significant genotype and amplitude interaction, p<0.0001). (D) Amplitude distribution analysis showed a significant increase in the percentage of small events (<10pA) and a trend towards a decrease in big events (>15pA) in Hu97/18 SPNs. (E) There was no difference in sEPSC decay time between Hu18/18 and Hu97/18 SPNs. (F) Cumulative probability showed an increase in sEPSC inter-event intervals in Hu97/18 SPNs (significant genotype and inter-event intervals interaction, p<0.0001). (G) The average sEPSC frequency was decreased in Hu97/18 SPNs (p = 0.038). For all experiments, Hu18/18 n = 20 and Hu97/18 n = 17. *P<0.05 (two-way ANOVA with Bonferroni correction, D; unpaired Student’s t-test, G).

To test for alterations in synaptic transmission, we recorded spontaneous excitatory postsynaptic currents (sEPSCs) at V_h_ = -70 mV in ACSF with the GABA_A_ channel blocker PTX (Fig. 2A). We found a significant decrease in the amplitude of sEPSCs in Hu97/18 SPNs as shown by the cumulative probability analysis (interaction p<0.0001, Fig. 2C), although the mean difference did not reach statistical significance (p = 0.15, Fig. 2B). A more detailed analysis of amplitude distribution revealed a higher percentage of small events (<10pA, p<0.01) as well as a trend to a lower percentage of large events (15–30pA) in Hu97/18 (Fig. 2D). These data suggest the possibility of a modest decrease in postsynaptic AMPA receptor number in striatal SPNs of Hu97/18 mice. In contrast, mean sEPSC charge transfer and kinetics were not significantly different between genotypes (for the decay time, see Fig. 1E; charge transfer and rise time not shown).

**Figure 3:**
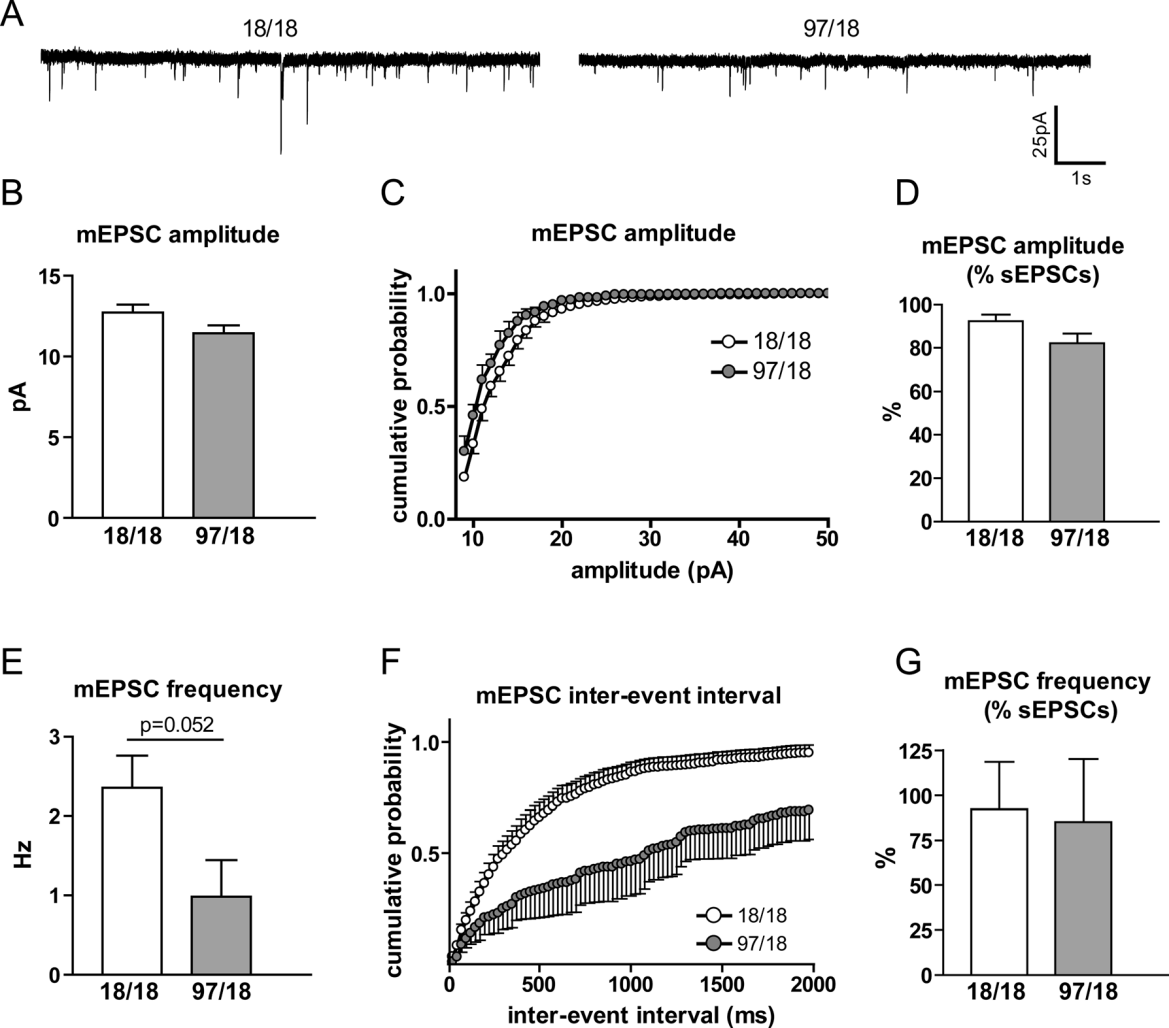
mEPSC amplitude and frequency change in Hu97/18 SPNs. Cells were patch-clamped with potassium-based internal solution at V_h_ = -70 mV and the initial sEPSCs were recorded. Then, TTX was added to the external solution which allowed for the extraction and analysis of mEPSCs. (A) Representative mEPSC traces for Hu18/18 (left) and Hu97/18 SPNs (right). (B) The average mEPSC amplitude of Hu18/18 and Hu97/18 cells (p = 0.1). (C) Cumulative probability showed a decrease in sEPSC amplitude in Hu97/18 SPNs (significant genotype and amplitude interaction, p<0.0001). (D) mEPSC amplitude presented as a percentage of the initial sEPSC amplitude. There was no difference between Hu18/18 and Hu97/18 SPNs. (E) The mean mEPSC frequency trended towards a decrease in Hu97/18 SPNs but did not reach statistical significance (p = 0.052). (F) Cumulative probability showed an increase in mEPSC inter-event intervals in Hu97/18 SPNs (significant genotype and inter-event intervals interaction, p<0.0001). (G) mEPSC frequency presented as a percentage of the initial sEPSC frequency. There was no difference between Hu18/18 and Hu97/18 SPNs. For all experiments, Hu18/18 n = 9 and Hu97/18 n = 5.

Analysis of the frequency of events revealed a significant reduction in Hu97/18 compared to Hu18/18 SPNs, as shown by both the cumulative probability of inter-event intervals (interaction p<0.0001, Fig. 2F) and the mean event frequency (p = 0.038, Fig. 2G). When TTX was included in the bath solution to block action potential-driven transmitter release, we found similar genotype effects on mEPSC frequency and amplitude as we did with sEPSCs (Fig. 3A–G). In fact, the application of TTX had very little effect on the amplitude (Fig. 3D) or frequency (Fig. 3G) of spontaneous events, confirming a previous report that the majority of spontaneous excitatory transmitter release in our preparation is action potential-independent [20].

**Figure 4:**
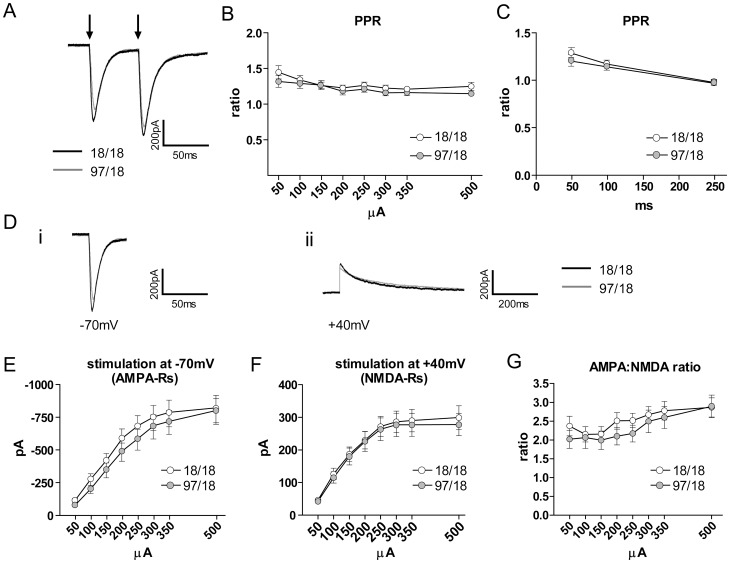
Paired-pulse ratio (PPR) and AMPA:NMDA ratio are not affected in Hu97/18 SPNs. SPNs were whole-cell patch-clamped at V_h_ = -70 mV with caesium-based internal solution to allow for better membrane voltage control. (A) Representative traces showing responses of Hu18/18 and Hu97/18 SPNs to 200 µA paired-pulse stimulation (marked with the arrows) with 50 ms interval. Stimulus artifacts were removed. (B–C) No difference between Hu18/18 and Hu97/18 SPNs in PPR with different stimulation intensities (50–500 µA; B) and different time of inter-stimulation intervals (50, 100 and 250 ms; C) implies no change in probability of release from excitatory presynaptic terminals. For the experiments in (B), Hu18/18 n = 22 and Hu97/18 n = 22; for the experiments in (C), Hu18/18 n = 11 and Hu97/18 n = 14. (D) Representative responses of Hu18/18 and Hu97/18 SPN to 200 µA stimulation at V_h_ = -70 mV (i), analysed as AMPA receptor response, and at V_h_ = +40 mV (ii), analysed as NMDA receptor response at 40 ms from the initial peak. Stimulus artifacts were removed. (E) There was no statistically significant difference in the response to the stimulation at V_h_ = -70 mV between Hu18/18 (n = 22) and Hu97/18 SPNs (n = 22). The trend towards a smaller response in Hu97/18 SPNs may suggest, however, a decreased number of synaptic AMPA receptors. (F) No difference in the response to the stimulation at V_h_ = +40 mV between Hu18/18 (n = 22) and Hu97/18 SPNs (n = 21) implies no change in NMDA receptor expression levels. (G) AMPA:NMDA ratios (measured as an average from the cells where both responses to the stimulation at V_h_ = -70 mV and V_h_ = +40 mV were recorded) showed no difference between Hu18/18 (n = 19) and Hu97/18 SPNs (n = 19).

The decreased sEPSC frequency is consistent with a loss of excitatory synapses on these cells, which is a signature mark of HD progression in other animal models [14], [15]. On the other hand, the decreased sEPSC frequency may be linked to changes in release probability from excitatory presynaptic terminals [16]. A decrease in release probability would lead to a decrease in the frequency of postsynaptic glutamate receptor activation, which would be detected as a lower number of sEPSC events, a change that has been reported in other HD models [12], [17], [18]. To assess the release probability from presynaptic excitatory terminals, we patched SPNs with a caesium-based internal solution and recorded responses to paired electrical stimulation of the nearby afferents (150–200 µm from the patch-pipette; Fig. 3A). The two consecutive stimulations (50 ms apart) allowed us to calculate paired-pulse ratio (PPR), measured as the response to the second stimulation divided by the response to the first. An increase in PPR (also known as paired-pulse facilitation) implies a lower initial probability of release, while a decrease (paired-pulse depression) indicates a higher probability. We used increasing stimulation intensities (50–500 µA) to detect any possible changes between the two genotypes that depend on the stimulus strength. At 9 months of age, no difference in PPR was observed between Hu18/18 and Hu97/18 SPNs, indicating no differences in the release probability from excitatory terminals (Fig. 4A,B). Moreover, at a stimulus intensity that generated an approximately half-maximal response, there was also no effect of genotype on PPRs with varying pulse intervals (Fig. 4C). Thus, despite a reduction in sEPSC frequency, we find no clear electrophysiological evidence of altered release probability.

The expression levels of AMPA receptors were previously shown to decrease with disease progression in a variety of HD mouse models [18], [19] while NMDA receptor levels have been reported as increased or decreased, depending on the HD mouse model, stage of disease, and subcellular localization of the receptors [14], [19], [20]. Our analysis of sEPSCs has suggested that the levels of synaptic AMPA receptors may be modestly decreased in Hu97/18 SPNs (Fig. 2C–D). To evaluate levels of synaptic NMDA receptors, we measured the ratio of evoked EPSCs (eEPSCs) at negative (-70 mV) and positive (+40 mV) potentials in the same cell. Current recorded at -70 mV reflects activation of AMPA receptors only (due to cells being held at negative potential and recorded in ACSF containing a physiological concentration of Mg^2+^, which prevents opening of NMDA receptors, and picrotoxin, which blocks GABA_A_ receptors), while responses at +40 mV, measured 40 ms following initial peak current, reflect activation of NMDA receptors. The AMPA:NMDA ratio was then calculated. While the mean evoked response size was slightly smaller in Hu97/18 SPNs at -70 mV (Fig. 4E), there was no significant genotype difference at this holding potential or at +40 mV (Fig. 4F). Similarly, the AMPA:NMDA ratio was unchanged (Fig. 4G).

### Progressive electrophysiological differences in striatal SPNs recorded from Hu97/18 and Hu18/18 mice

After characterising electrophysiological changes in Hu97/18 SPNs at 9 months of age, we investigated whether the same alterations could be observed at an earlier time point (3 and 6 months) or rather if they represent progressive deficits. At 6 months of age, Hu97/18 mice already demonstrate many of the behavioral and cognitive deficits that become more pronounced at 9 months [11], and therefore we expected to see some, if not all, of the electrophysiological changes that we recorded in older animals.

**Figure 5:**
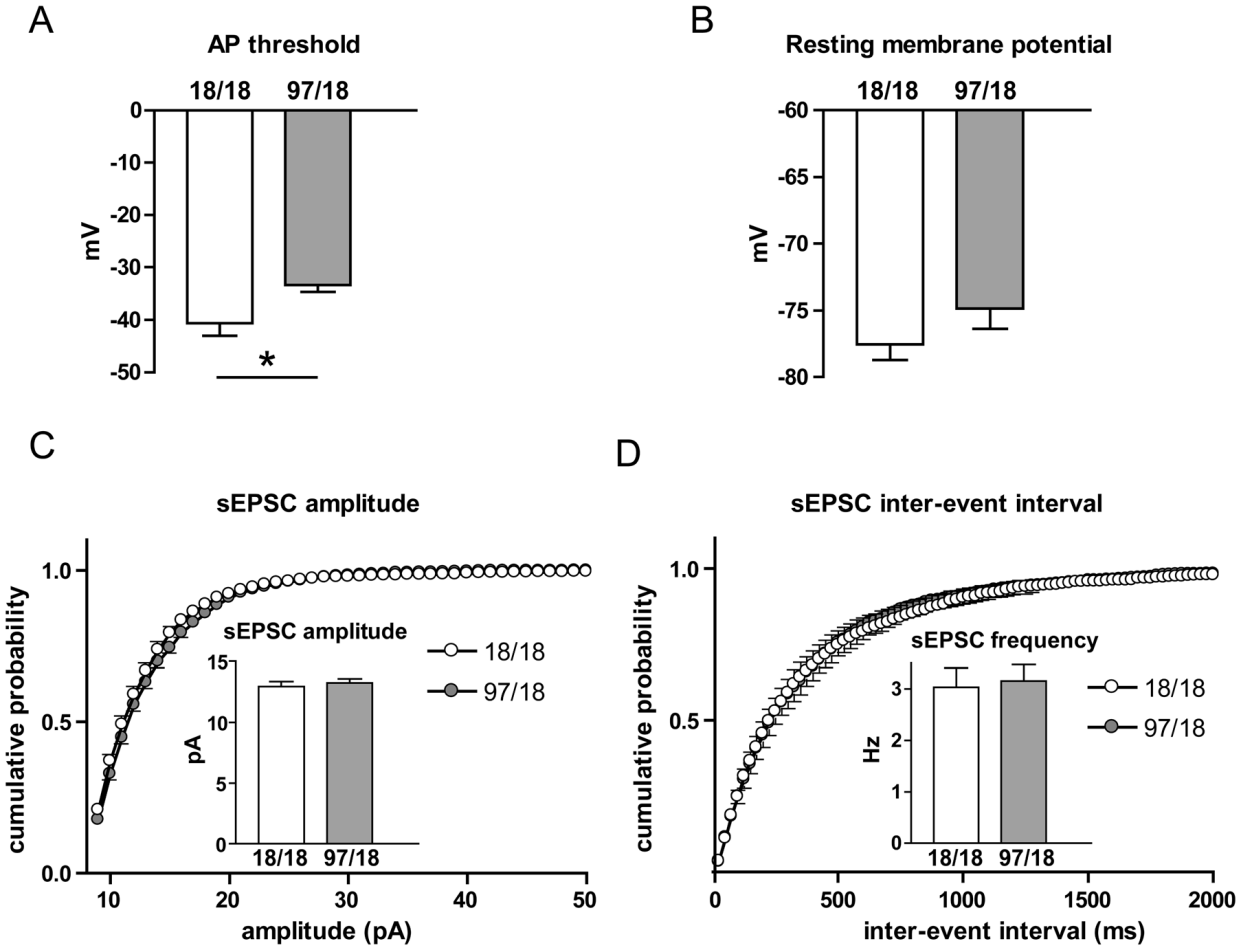
Subtle changes in membrane properties and unaltered sEPSC characteristics in Hu97/18 SPNs at 6 months of age. (A) At 6 months of age, action potential threshold was more depolarized in Hu97/18 SPNs. (B) No significant change in resting membrane potential was observed. sEPSC amplitude (C) and frequency (D) were unaffected in Hu97/18 SPNs at 6 months of age. For these experiments, Hu18/18 n = 13 and Hu97/18 n = 16. *p<0.05 (unpaired Student’s t-test).

**Figure 6:**
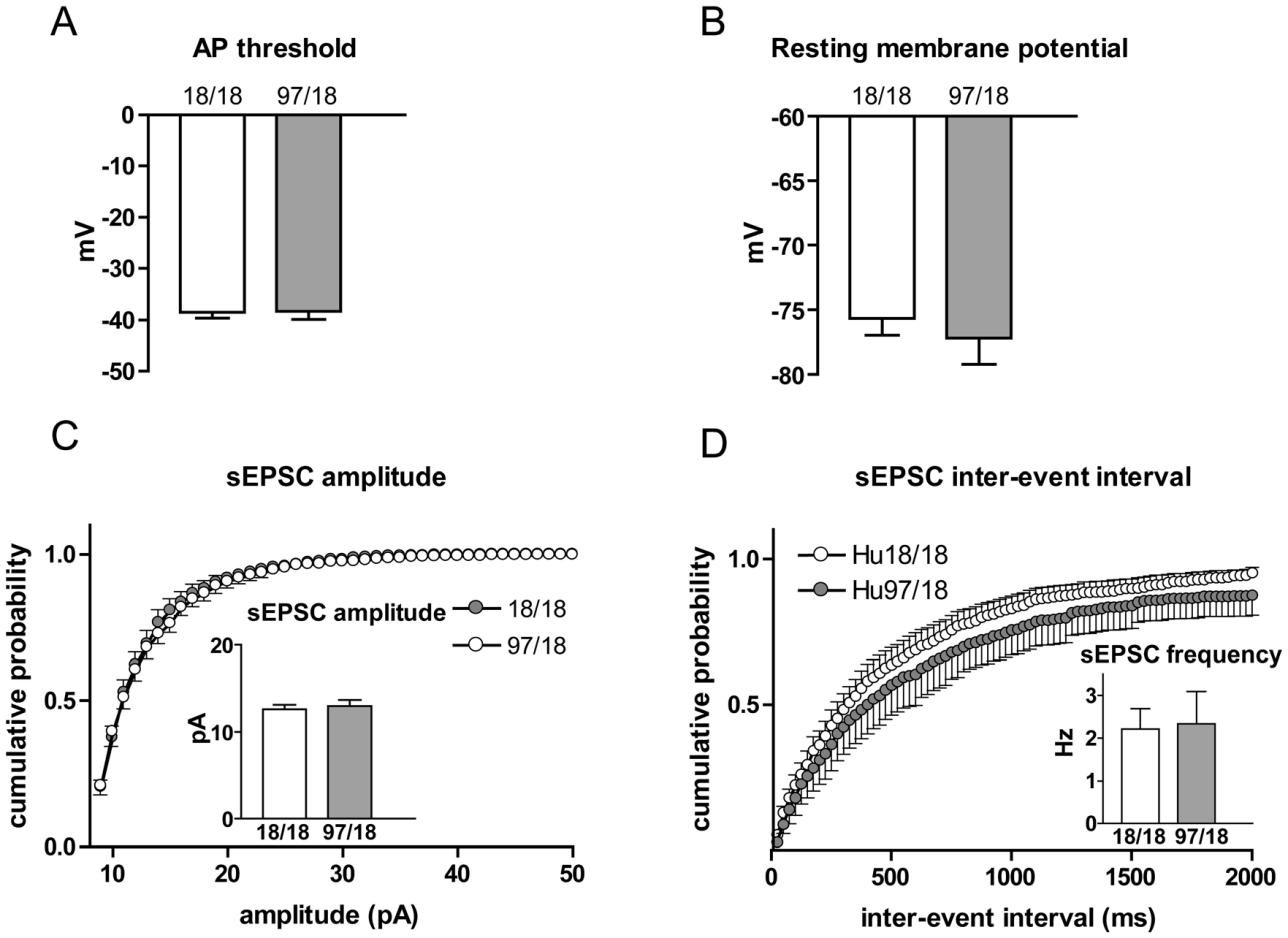
No changes in membrane properties and sEPSC characteristics in Hu97/18 SPNs at 3 months of age. At 3 months of age, action potential threshold (A) and resting membrane potential (B) were unaltered in Hu97/18 (n = 10), in comparison to Hu18/18 SPNs (n = 11). Likewise, sEPSC amplitude (C) and frequency (D) were unaffected in Hu97/18 (n = 8) when compared to Hu18/18 SPNs (n = 8).

Similar to mice at 9 months of age, we observed no difference in membrane capacitance, resistance or tau. I–V curves, rheobase and rheobase frequency were also similar between Hu18/18 and Hu97/18 SPNs at 6 months of age (data not shown). However, the action potential threshold was increased (p = 0.02, Fig. 5A), similar to the results obtained from 9 month-old animals (Fig. 1I), while depolarization of the resting membrane potential appeared as a trend, but did not reach statistical significance (p = 0.18, Fig. 5B). Unlike at 9 months, there was no detectable difference in sEPSC frequency or amplitude at 6 months (Fig. 5C–D). When we examined an even earlier time point (3 months), we were unable to detect any difference in action potential threshold, resting membrane potential, sEPSC frequency or amplitude (Fig. 6 A–D). Together, these data suggest that the changes observed in Hu97/18 SPNs at 9 months of age represent progressive characteristics, and that the majority of these changes arise between 6 and 9 months of age.

### Impaired hippocampal CA1 long-term potentiation in Hu97/18 mice

**Figure 7:**
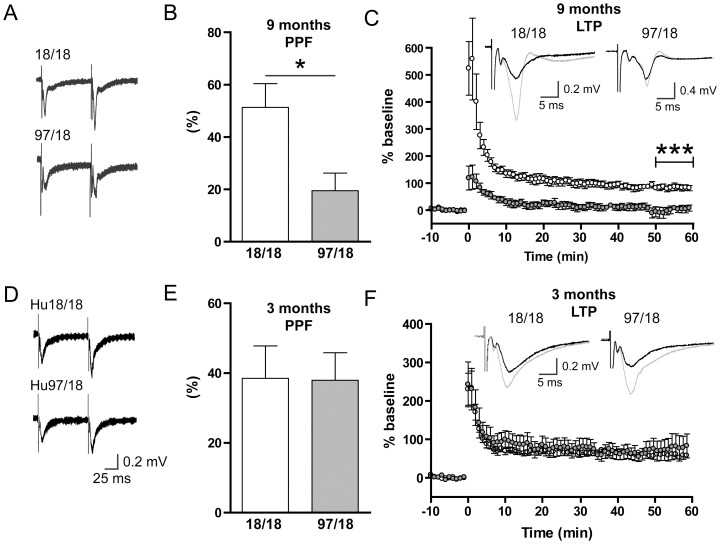
Long-term potentiation (LTP) is impaired in 9-month old Hu97/18 CA1. (A) Representative fEPSP traces from hippocampal slices of 9-month old Hu18/18 and Hu97/18 mice in response to two 0.1 ms pulses separated by 100 ms. fEPSPs were recorded in CA1 stratum radiatum during stimulation of the Schaffer collateral pathway. (B) Paired pulse facilitation (PPF) was measured by dividing the slope of the second response to that of the first and was expressed as percent increase. PPF was significantly lower in Hu97/18 CA1 (Hu18/18 n = 8, Hu97/18 n = 6). (C) fEPSPs were normalized to a 10-minute baseline period prior to high-frequency stimulation (HFS; 100 Hz for 1 s×3, 10 s inter-train interval). LTP was easily obtained in hippocampal slices from Hu18/18 mice (n = 6) but showed severe impairment in slices from Hu97/18 mice (n = 6). Representative traces before (black) and 50–60 minutes after (grey) HFS are shown. ***p<0.0001, t-test of average % above baseline 50–60 minutes post-HFS. (D–F) Paired pulse facilitation (D,E) and LTP (F) graphs as above but conducted in slices from 3-month old animals. n = 6 for each genotype for both (E) and (F).

**Figure 8:**
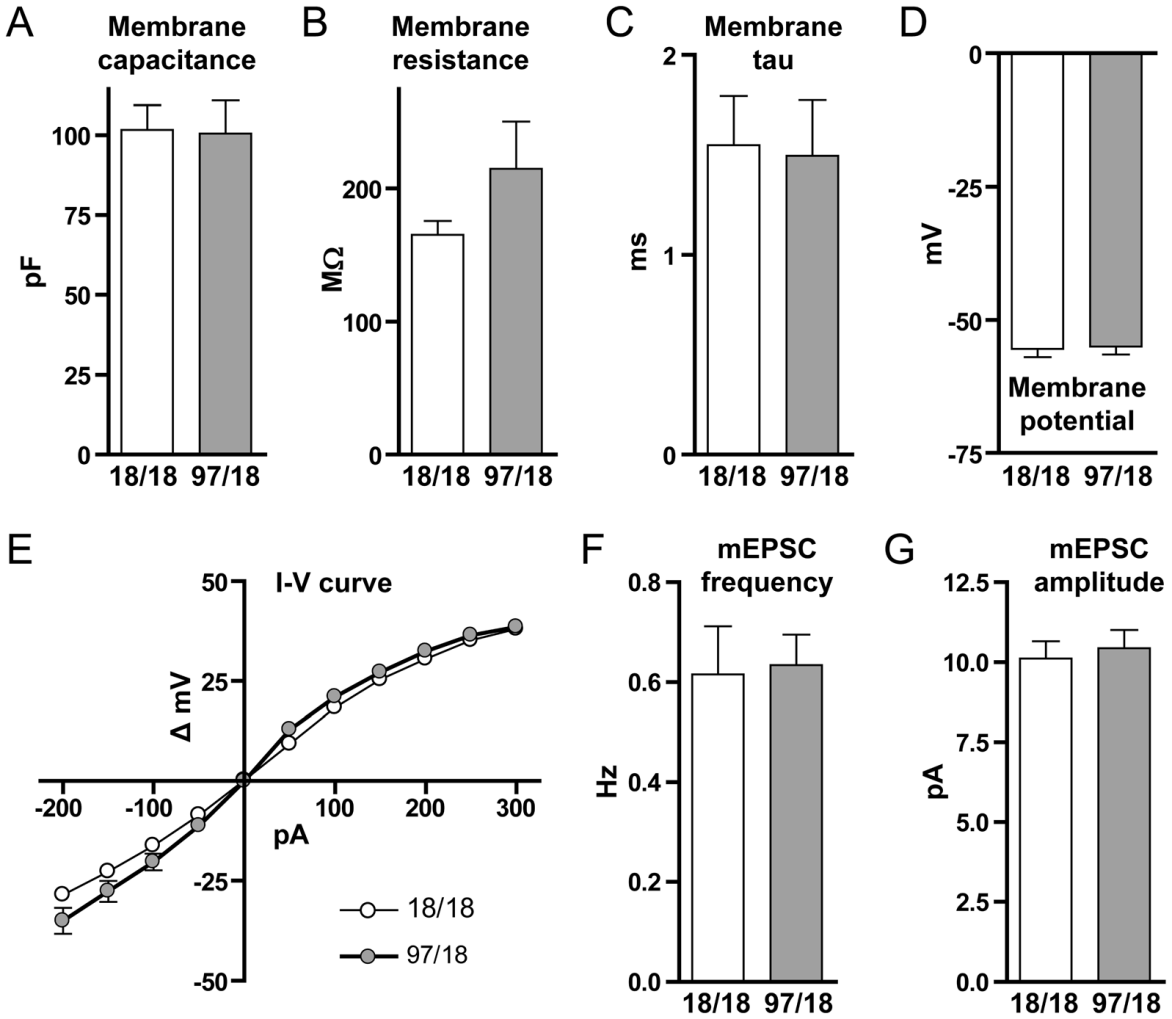
Whole-cell properties of CA1 pyramidal neurons from Hu18/18 and Hu97/18 mice at 9 months of age. (A–D) Whole-cell patch recordings from CA1 pyramidal neurons revealed no difference in membrane properties including membrane capacitance (Cm, Hu18/18 n = 14, Hu97/18 n = 11), membrane resistance (Rm, Hu18/18 n = 14, Hu97/18 n = 11), membrane tau (ôm, Hu18/18 n = 14, Hu97/18 n = 11) or resting membrane potential (Em, Hu18/18 n = 14, Hu97/18 n = 13). I–V plots (E, Hu18/18 n = 14, Hu97/18 n = 13), mEPSC frequency (F, Hu18/18 n = 13, Hu97/18 n = 13) and mEPSC amplitude (G, Hu18/18 n = 13, Hu97/18 n = 13) were also similar between genotypes.

At 9 months, Hu97/18 mice display extensive deficits in learning and memory performance, as shown by spatial learning and novel object recognition tests [11]. To determine whether these behavioral changes may stem from deficits in hippocampal long-term potentiation (LTP), as shown previously in other mouse models of HD [21]–[24], we stimulated excitatory field potentials (fEPSPs) in CA1 stratum radiatum. First, using a standard paired-pulse paradigm (at an interval of 100 ms), we found significantly reduced paired-pulse facilitation (PPF) in Hu97/18 slices (Fig. 7A–B; Hu18/18 n = 8, Hu97/18 n = 6, p = 0.021), indicating an impairment in short-term plasticity and consistent with a previous report in another HD mouse model [22]. We also observed a severe deficit in LTP in hippocampal slices from Hu97/18 mice (Fig. 7C). The presence of robust potentiation 50–60 minutes following high-frequency stimulation (HFS) in slices from Hu18/18 mice (n = 6) demonstrates that the replacement of murine HTT with human HTT does not impair LTP. On the other hand, LTP was completely absent in slices from Hu97/18 mice (n = 6, p = 0.0005 for Hu18/18 vs. Hu97/18, unpaired t-test of average fEPSP slope 50–60 minutes post-HFS). In contrast, paired pulse facilitation was normal and LTP was intact in slices from 3-month old Hu97/18 mice (Fig. 7D–F). Lastly, when we recorded CA1 pyramidal neurons under whole cell voltage-clamp at 9 months of age, we were unable to detect any genotype differences in membrane properties or mEPSC frequency or amplitude (Fig. 8A–G), demonstrating that alterations in these cellular and synaptic properties are at least somewhat specific to SPNs at this age. In all, our data support a progressive hippocampal synaptic plasticity deficit in HD and, at 9 months of age, provide an additional robust electrophysiological phenotype in the humanized mouse model of HD.

## Discussion

Here, we examined for the first time the electrophysiological changes that accompany behavioral and neuropathological deficits reported in the Hu97/18 mouse model [11]. Hu97/18 is a novel and unique mouse model of HD that fully recapitulates the genetics of the human disease by expressing human huntingtin heterozygous for an expanded polyglutamine tract (97 repeats) on a mouse huntingtin *null* (*Hdh^-/-^*) background [11]. We describe many electrophysiological alterations in Hu97/18 SPNs, such as progressive changes in membrane properties and synaptic transmission, as well as a progressive impairment of synaptic plasticity in the hippocampus. These robust electrophysiological measures, along with the known behavioral and neuropathological changes [11], can serve as future tools to assess the efficacy of muHTT-specific ASOs [8] and other promising therapeutic options to prevent or reverse the detrimental effects of muHTT on CNS function.

### Alterations in cellular and synaptic properties of striatal spiny projection neurons

Membrane capacitance, membrane resistance and tau were not altered in Hu97/18 SPNs at 6 or 9 months of age. This is not surprising, given the fact that these features are not altered in other slowly-progressing mouse models, such as YAC128 or CAG140, until 12 months of age [25]. Earlier changes in membrane resistance were seen in the more rapidly-progressing R6/2 model, which recapitulates more accurately the phenotype of juvenile HD [17], [26].

The depolarized resting membrane potential that we observed in Hu97/18 SPNs has also been noted for other HD mouse models [27], [28]. It has been hypothesized that, together with large discharges from cortical afferents (detected as large, >100pA sEPSCs: [17]) and elevated input resistance, this change would directly lead to an increase in SPN excitability (as detected *in vivo*: [29]). However, Hu97/18 SPNs did not display changes in membrane resistance, nor did we observe any large-amplitude sEPSCs. Also, the current-voltage step protocol did not detect any changes in rheobase or rheobase frequency between Hu97/18 and Hu18/18 SPNs, indicating that the propagation of a signal requires similar (and not lower) stimulus intensity. This is most probably achieved by a homeostatic mechanism that involves an increase in action potential threshold. This further strengthens our conclusion that despite small changes in membrane characteristics (e.g., the depolarized resting membrane potential), Hu97/18 SPNs are not more excitable than Hu18/18 SPNs.

At 9 months of age, the sEPSC and mEPSC amplitudes were decreased in comparison to Hu18/18 SPNs, suggesting a lower number of AMPA receptors in synapses. Similar results have been previously reported in YAC128 at 1 month of age [12], but also at 7 months of age with evoked EPSCs [18] and in symptomatic R6/2 mice with AMPA bath application [19]. Therefore, this effect does not seem to be restricted to a fully symptomatic stage of HD, but more likely depends on the model of animal used and its genetic background. The Hu97/18 model is derived from BACHD mice that show changes in sEPSC amplitude at 6 months of age [30]. However, Hu97/18 mice express ∼40% less muHTT than the BACHD mice [11] and so it is not surprising that the changes in sEPSCs that we observed at 9 months of age were not apparent at 6 months.

Another prominent alteration in mEPSCs and sEPSCs recorded from Hu97/18 SPNs was a decrease in frequency, which is a common change observed in HD mouse models at symptomatic stages of the disease [17], [25]. This is often associated with a reduced dendritic arborization and loss of dendritic spines and excitatory synapses, both in animal models of HD [14], [17], [28] and in human HD patients [14]. In the present study, it was a little surprising that we did not observe a significant decrease in the size of AMPAR-mediated eEPSCs at -70 mV (see Fig. 4E), which would be expected following synaptic loss. However, the response magnitude in these experiments relies largely on electrode placement and can vary greatly from one experiment to the next. The mean eEPSC amplitude tended to be lower in Hu97/18 SPNs (see Fig. 4E); however, response size variability was too high to observe significant differences between genotypes. The alternative explanation, a decrease in release probability from presynaptic terminals, was ruled out based on no change in the paired-pulse ratio of synaptically-evoked responses.

The lack of alteration in release probability from excitatory terminals, together with the lack of large sEPSCs (suggested by others to be action-potential driven: [17]) is interesting, as it suggests no significant changes in intact corticostriatal afferents. In presymptomatic YAC72 and YAC128 mice, the release probability is significantly decreased ([12]; also observed in symptomatic YAC128: [18]), while it has been shown to be increased in pre- and symptomatic R6/2 mice [28]. Also, marked changes have been described in recordings from cortical cells from symptomatic R6/2 mice, including changes in basic membrane properties, sEPSC frequency and appearance of complex discharges [31]. Therefore, it would be interesting to examine the possibility of such changes occurring in cortical neurons from the Hu97/18 mouse model; however, these experiments are beyond the scope of this particular study.

Our finding that synaptic AMPAR current amplitude is reduced (based on sEPSC amplitude distribution), while the ratio of evoked AMPAR to NMDAR EPSC amplitude is similar between genotypes, suggests that the number of synaptic NMDA receptors may also be decreased at 9 months of age. This possibility is not entirely surprising, as although NMDA:AMPA ratio has been reported to be increased in presymptomatic YAC72 and YAC128 mice compared to controls [12], [32], at symptomatic stages YAC128 SPNs display a decrease in NMDA currents, accompanied by resistance to neurotoxicity [14]. This change could be attributed to an increase in the removal/degradation rate of NMDA receptors on the surface of SPNs [33], as well as to a mislocalization of the receptors from synaptic to extrasynaptic sites [20], [34] due to an increase in calpain and STEP61 activity [33], [34].

### Impaired hippocampal long-term potentiation

We also recorded field potentials in CA1 stratum radiatum and found a complete absence of LTP in slices from 9-month old Hu97/18 mice 50–60 minutes following HFS. This finding is consistent with other mouse models of HD [21]–[24] and provides a mechanistic basis for the spatial learning deficit observed previously in Hu97/18 mice [11]. Interestingly, this learning deficit was progressive in that no impairment was seen at 3 months of age [11], a time at which we show that LTP is intact. While the molecular mechanisms underlying the observed LTP deficit at 9 months were not explored in the present study, previous work demonstrated that the LTP deficit in a knock-in mouse model of HD was restored by BDNF application directly to the slice [24] or by inducing BDNF upregulation through daily injections of an ampakine [35]. This is consistent with a role for BDNF/TrkB signalling in hippocampal plasticity [36], as well as muHTT’s inhibitory effect on BDNF production [37]. The cognitive decline associated with HD is highly debilitating and can manifest many years before the onset of cell death and overt motor symptoms. With robust and progressive deficits in both LTP and spatial memory, the Hu97/18 model is particularly suited for preclinical assessment of the effects of early treatment interventions on cognitive decline in HD.

### Different genetic backgrounds of HD models and their consequences

As we have noted above, there are many similarities between the changes in Hu97/18 SPNs and those reported in other animal models. However, there are also surprising differences, such as unaltered physiology of glutamatergic input release probability and no change in SPN action potential firing properties. Furthermore, while many cellular and synaptic alterations have been reported to occur prior to measurable behavioral abnormalities, we were unable to detect any robust electrophysiological phenotype in the striatum at 6 months of age, a stage when many cognitive and motor impairments are evident. It is possible that other measures not assessed in the present study, including the extrasynaptic NMDA receptor currents [38], [20], are indeed altered early in the Hu97/18 model. Nonetheless, we were surprised by the lack of electrophysiological effects at 6 months of age, and our data suggest that the bulk of synaptic deficits in the striatum occur between 6 and 9 months of age. These discrepancies may primarily stem from a different genetic background of multiple models that leads to differences in the number of CAG repeats, or muHTT expression patterns and levels (reviewed in: [9], [10]; see also: [11], [25], [27], [39], [40]). BACHD mice, which the Hu97/18 model is largely based on, have not been fully described using electrophysiological methods [30]. Also, a direct comparison between BACHD and Hu97/18 could be misleading, as BACHD expresses normal levels of mouse HTT (on top of human muHTT) and its levels of muHTT are ∼40% higher than those of Hu97/18 [11].

## Conclusion

The Hu97/18 model is unique as it is the first animal model to fully recapitulate the genetics of human HD. It is critically important to track the changes in its phenotype, not only to expand our knowledge of HD and its progression, but also for the application and assessment of therapies in the future.

## References

[1] The Huntington’s Disease Collaborative Research Group (1993) A novel gene containing a trinucleotide repeat that is expanded and unstable on Huntington’s disease chromosomes. Cell 72: 971–983. 8458085 10.1016/0092-8674(93)90585-e

[2] Zuccato C, Valenza M, Cattaneo E (2010) Molecular mechanisms and potential therapeutical targets in Huntington’s disease. Physiol Rev 90: 905–981. 20664076 10.1152/physrev.00041.2009

[3] Di Filippo M, Tozzi A, Picconi B, Ghiglieri V, Calabresi P (2007) Plastic abnormalities in experimental Huntington’s disease. Curr Opin Pharmacol 7: 106–111. 17071137 10.1016/j.coph.2006.08.010

[4] Raymond LA, André VM, Cepeda C, Gladding CM, Milnerwood AJ, et al (2011) Pathophysiology of Huntington’s disease: time-dependent alterations in synaptic and receptor function. Neuroscience 198: 252–273. 21907762 10.1016/j.neuroscience.2011.08.052 PMC3221774

[5] Ghiglieri V, Bagetta V, Calabresi P, Picconi B (2012) Functional interactions within striatal microcircuit in animal models of Huntington’s disease. Neuroscience 211: 165–184. 21756979 10.1016/j.neuroscience.2011.06.075

[6] Southwell AL, Skotte NH, Bennett CF, Hayden MR (2012) Antisense oligonucleotide therapeutics for inherited neurodegenerative diseases. Trends Mol Med 18: 634–643. 23026741 10.1016/j.molmed.2012.09.001

[7] Martinez T, Wright N, López-Fraga M, Jiménez AI, Pañeda C (2013) Silencing human genetic diseases with oligonucleotide-based therapies. Hum Genet 132: 481–493. 23494242 10.1007/s00439-013-1288-1

[8] Carroll JB, Warby SC, Southwell AL, Doty CN, Greenlee S, et al (2011) Potent and selective antisense oligonucleotides targeting single-nucleotide polymorphisms in the Huntington disease gene / allele-specific silencing of mutant huntingtin. Mol Ther 19: 2178–2185. 21971427 10.1038/mt.2011.201 PMC3242664

[9] Cepeda C, Cummings DM, André VM, Holley SM, Levine MS (2010) Genetic mouse models of Huntington’s disease: focus on electrophysiological mechanisms. ASN Neuro 2: e00033. 20396376 10.1042/AN20090058 PMC2850512

[10] Pouladi MA, Morton AJ, Hayden MR (2013) Choosing an animal model for the study of Huntington’s disease. Nat Rev Neurosci 14: 708–721. 24052178 10.1038/nrn3570

[11] Southwell AL, Warby SC, Carroll JB, Doty CN, Skotte NH, et al (2013) A fully humanized transgenic mouse model of Huntington disease. Hum Mol Genet 22: 18–34. 23001568 10.1093/hmg/dds397 PMC3606012

[12] Milnerwood AJ, Raymond LA (2007) Corticostriatal synaptic function in mouse models of Huntington’s disease: early effects of huntingtin repeat length and protein load. J Physiol 585: 817–831. 17947312 10.1113/jphysiol.2007.142448 PMC2375504

[13] Kiraly DD, Lemtiri-Chlieh F, Levine ES, Mains RE, Eipper BA (2011) Kalirin binds the NR2B subunit of the NMDA receptor, altering its synaptic localization and function. J Neurosci 31: 12554–12565. 21880917 10.1523/JNEUROSCI.3143-11.2011 PMC3172699

[14] Graham RK, Pouladi MA, Joshi P, Lu G, Deng Y, et al (2009) Differential susceptibility to excitotoxic stress in YAC128 mouse models of Huntington disease between initiation and progression of disease. J Neurosci 29: 2193–2204. 19228972 10.1523/JNEUROSCI.5473-08.2009 PMC2729178

[15] Singaraja RR, Huang K, Sanders SS, Milnerwood AJ, Hines R, et al (2011) Altered palmitoylation and neuropathological deficits in mice lacking HIP14. Hum Mol Genet 20: 3899–3909. 21775500 10.1093/hmg/ddr308 PMC3177655

[16] Thomson AM (2000) Facilitation, augmentation and potentiation at central synapses. Trends Neurosci 23: 305–312. 10856940 10.1016/s0166-2236(00)01580-0

[17] Cepeda C, Hurst RS, Calvert CR, Hernández-Echeagaray E, Nguyen OK, et al (2003) Transient and progressive electrophysiological alterations in the corticostriatal pathway in a mouse model of Huntington’s disease. J Neurosci 23: 961–969. 12574425 10.1523/JNEUROSCI.23-03-00961.2003 PMC6741903

[18] Joshi PR, Wu NP, André VM, Cummings DM, Cepeda C, et al (2009) Age-dependent alterations of corticostriatal activity in the YAC128 mouse model of Huntington disease. J Neurosci 29: 2414–2427. 19244517 10.1523/JNEUROSCI.5687-08.2009 PMC2670193

[19] Cepeda C, Ariano MA, Calvert CR, Flores-Hernández J, Chandler SH, et al (2001) NMDA receptor function in mouse models of Huntington disease. J Neurosci Res 66: 525–539. 11746372 10.1002/jnr.1244

[20] Milnerwood AJ, Gladding CM, Pouladi MA, Kaufman AM, Hines RM, et al (2010) Early increase in extrasynaptic NMDA receptor signaling and expression contributes to phenotype onset in Huntington’s disease mice. Neuron 65: 178–190. 20152125 10.1016/j.neuron.2010.01.008

[21] Hodgson JG, Agopyan N, Gutekunst CA, Leavitt BR, LePiane F, et al (1999) A YAC mouse model for Huntington’s disease with full-length mutant huntingtin, cytoplasmic toxicity, and selective striatal neurodegeneration. Neuron 23: 181–192. 10402204 10.1016/s0896-6273(00)80764-3

[22] Usdin MT, Shelbourne PF, Myers RM, Madison DV (1999) Impaired synaptic plasticity in mice carrying the Huntington’s disease mutation. Hum Mol Genet 8: 839–846. 10196373 10.1093/hmg/8.5.839

[23] Murphy KP, Carter RJ, Lione LA, Mangiarini L, Mahal A, et al (2000) Abnormal synaptic plasticity and impaired spatial cognition in mice transgenic for exon 1 of the human Huntington’s disease mutation. J Neurosci 20: 5115–5123. 10864968 10.1523/JNEUROSCI.20-13-05115.2000 PMC6772265

[24] Lynch G, Kramar EA, Rex CS, Jia Y, Chappas D, et al (2007) Brain-derived neurotrophic factor restores synaptic plasticity in a knock-in mouse model of Huntington’s disease. J Neurosci 27: 4424–4434. 17442827 10.1523/JNEUROSCI.5113-06.2007 PMC6672319

[25] Cummings DM, Cepeda C, Levine MS (2010) Alterations in striatal synaptic transmission are consistent across genetic mouse models of Huntington’s disease. ASN Neuro 2: e00036. 20585470 10.1042/AN20100007 PMC2888168

[26] Ariano MA, Cepeda C, Calvert CR, Flores-Hernández J, Hernández-Echeagaray E, et al (2005) Striatal potassium channel dysfunction in Huntington’s disease transgenic mice. J Neurophysiol 93: 2565–2574. 15625098 10.1152/jn.00791.2004

[27] Levine MS, Klapstein GJ, Koppel A, Gruen E, Cepeda C, et al (1999) Enhanced sensitivity to N-methyl-D-aspartate receptor activation in transgenic and knockin mouse models of Huntington’s disease. J Neurosci Res 58: 515–532. 10533044

[28] Klapstein GJ, Fisher RS, Zanjani H, Cepeda C, Jokel ES, et al (2001) Electrophysiological and morphological changes in striatal spiny neurons in R6/2 Huntington’s disease transgenic mice. J Neurophysiol 86: 2667–2677. 11731527 10.1152/jn.2001.86.6.2667

[29] Rebec GV, Conroy SK, Barton SJ (2006) Hyperactive striatal neurons in symptomatic Huntington R6/2 mice: variations with behavioral state and repeated ascorbate treatment. Neuroscience 137: 327–336. 16257492 10.1016/j.neuroscience.2005.08.062

[30] Gray M, Shirasaki DI, Cepeda C, André VM, Wilburn B, et al (2008) Full-length human mutant huntingtin with a stable polyglutamine repeat can elicit progressive and selective neuropathogenesis in BACHD mice. J Neurosci 28: 6182–6195. 18550760 10.1523/JNEUROSCI.0857-08.2008 PMC2630800

[31] Cummings DM, André VM, Uzgil BO, Gee SM, Fisher YE, et al (2009) Alterations in cortical excitation and inhibition in genetic mouse models of Huntington’s disease. J Neurosci 29: 10371–10386. 19692612 10.1523/JNEUROSCI.1592-09.2009 PMC2754238

[32] Li L, Murphy TH, Hayden MR, Raymond LA (2004) Enhanced striatal NR2B-containing N-methyl-D-aspartate receptor-mediated synaptic currents in a mouse model of Huntington disease. J Neurophysiol 92: 2738–2746. 15240759 10.1152/jn.00308.2004

[33] Cowan C, Fan MM, Fan J, Shehadeh J, Zhang LY, et al (2008) Polyglutamine-modulated striatal calpain activity in YAC transgenic huntington disease mouse model: impact on NMDA receptor function and toxicity. J Neurosci 28: 12725–12735. 19036965 10.1523/JNEUROSCI.4619-08.2008 PMC6671821

[34] Gladding CM, Sepers MD, Xu J, Zhang LY, Milnerwood AJ, et al (2012) Calpain and STriatal-Enriched protein tyrosine phosphatase (STEP) activation contribute to extrasynaptic NMDA receptor localization in a Huntington’s disease mouse model. Hum Mol Genet 21: 3739–3752. 22523092 10.1093/hmg/dds154 PMC3412376

[35] Simmons DA, Rex CS, Palmer L, Pandyarajan V, Fedulov V, et al (2009) Up-regulating BDNF with an ampakine rescues synaptic plasticity and memory in Huntington’s disease knockin mice. Proc Natl Acad Sci USA 106: 4906–4911. 19264961 10.1073/pnas.0811228106 PMC2660722

[36] Minichiello L (2009) TrkB signalling pathways in LTP and learning. Nat Rev Neurosci 10: 850–860. 19927149 10.1038/nrn2738

[37] Zuccato C, Ciammola A, Rigamonti D, Leavitt BR, Goffredo D, et al (2001) Loss of huntingtin-mediated BDNF gene transcription in Huntington’s disease. Science 293: 493–498. 11408619 10.1126/science.1059581

[38] Okamoto S, Pouladi MA, Talantova M, Yao D, Xia P, et al (2009) Balance between synaptic versus extrasynaptic NMDA receptor activity influences inclusions and neurotoxicity of mutant huntingtin. Nat Med 15: 1407–1413. 19915593 10.1038/nm.2056 PMC2789858

[39] Cummings DM, Alaghband Y, Hickey MA, Joshi PR, Hong SC, et al (2012) A critical window of CAG repeat-length correlates with phenotype severity in the R6/2 mouse model of Huntington’s disease. J Neurophysiol 107: 677–691. 22072510 10.1152/jn.00762.2011 PMC3349621

[40] Pouladi MA, Stanek LM, Xie Y, Franciosi S, Southwell AL, et al (2012) Marked differences in neurochemistry and aggregates despite similar behavioural and neuropathological features of Huntington disease in the full-length BACHD and YAC128 mice. Hum Mol Genet 21: 2219–2232. 22328089 10.1093/hmg/dds037

